# Superior mesenteric vein injury in penetrating abdominal trauma: A case report

**DOI:** 10.1016/j.ijscr.2018.09.040

**Published:** 2018-09-29

**Authors:** Nada Faris Alhassan, Saad Mohammed Alsaawy, Ibrahim Tawfiq Albabtain

**Affiliations:** a College of Medicine, King Saud bin Abdul-Aziz University for Health Sciences, P.O Box 22490, 11426, Riyadh, Saudi Arabia; b Prince Sattam bin Abdulaziz University, Al-kharj, Saudi Arabia; c Department of General Surgery, King Abdul-Aziz Medical City, Ministry of National Guard Health Affairs, Riyadh, Saudi Arabia

**Keywords:** Care report, superior mesenteric vein, Inferior vena cava, Trauma, Abdominal vascular injury, Gunshot

## Abstract

**Introduction:**

Injury to the superior mesenteric vein (SMV) is considerably rare amongst abdominal visceral vascular injuries. Multiple factors play a role in identifying such injuries, leading to the high morbidity and mortality associated.

**Case presentation:**

We report a twenty-six-year-old male sustaining a SMV injury following a self-inflected gun shot. Initial assessment of the patient reveals hemodynamic instability and intraabdominal hemorrhage. Surgically approaching the patient was performed in two stages. Midline laparotomy was performed to control the active bleeding and stabilize the patient. Kocherizing the duodenum reveals that the source of retroperitoneal bleeding was from the SMV and one of the supra-renal tributaries of the Inferior Vena Cava (IVC). Several stitches were taken in a figure-of-eight manner to control the bleeding. Second-look operation was performed to rule out other injuries and permeant closure of the abdominal wall. Proper psychiatric care was ensured before transferal of the patient in good conditions to another healthcare facility specialized in mental health.

**Discussion:**

High mortality rates reaching up to 65% are reported in similar cases. Multiple factors have contributed to the patient’s favorable outcome, including rapid trauma response, absences of other associated injuries, and the patient’s baseline healthy status.

**Conclusion:**

The principle of damage control surgery in trauma is an effective strategy to stabilize the patient and rule out other injuries which might not have been discernible initially.

## Highlights

• Superior mesenteric vein and inferior vena cava injuries are associated with high mortality.
• Damage control surgery is an effective strategy to stabilize the patient initially.
• Second-look operations allow to identify injuries which might not have been discernable at first.
• A mature hospital system with rapid response to trauma is integral when handling gunshot injuries.

## 1 Introduction

Abdominal visceral vascular injuries are responsible for only 0.1–1% of overall vascular trauma, making them very uncommon, with superior mesenteric vein (SMV) injuries being even more rare [1]. The control and repair of Intra-abdominal vascular injuries pose a considerable challenge due to several factors. Those include the technicality of the surgery itself, owing to the subsequent massive bleeding, difficult exposure, associated multiple injuries, different methods of surgical repair, and the need for second-look operations [2]. Intra-abdominal vascular injuries are associated high morbidity and mortality. SMV injuries carry significant mortality rates, ranging from 45% to 71% [[2], [3], [4]]. Associated deaths can be either due to the massive hemorrhagic shock, or delayed, after surviving the surgical repair; mainly secondary to sepsis and subsequent small bowel ischemia [5]. Through recent years, improvements in health care system that include better transportation, prompt resuscitation and surgical interventions, have played a role in decreasing mortality rates of those injuries [3]. Herein, we report a case of SMV injury sustained after penetrating gunshot trauma that was successfully treated with primary surgical repair. This work has been reported in line with the SCARE criteria [6].

## 2 Case presentation

**Fig. 1:**
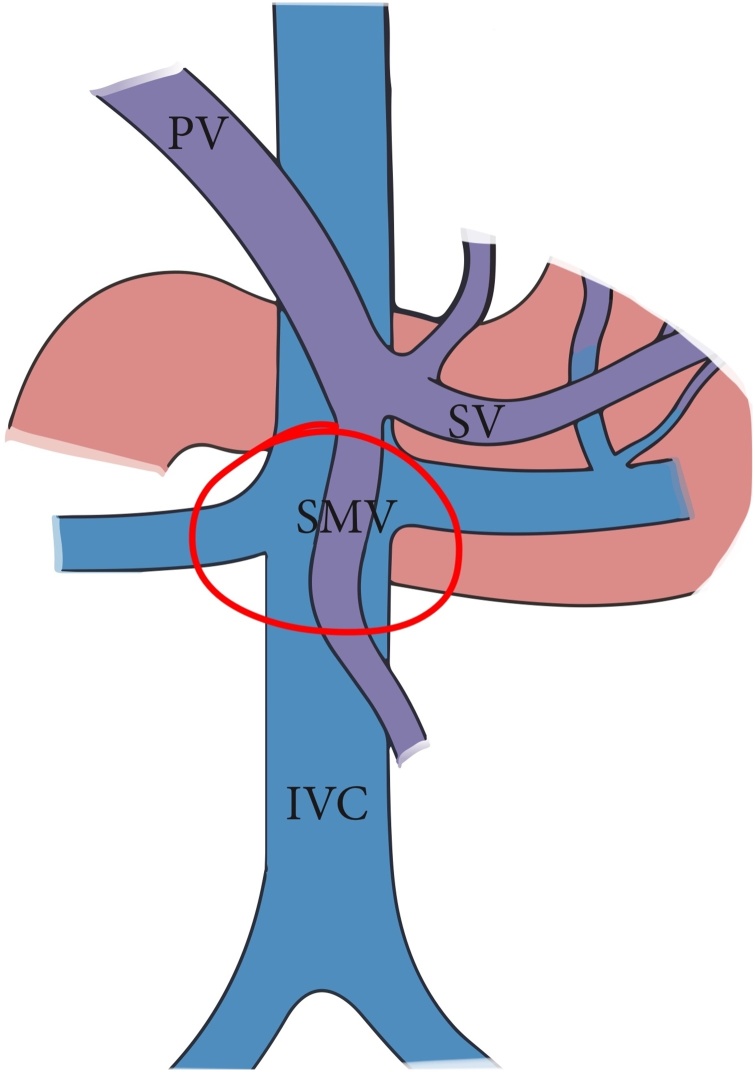
Basic anatomy of the portosystemic circulation. Red circle delineates the site of injury.

**Fig. 2:**
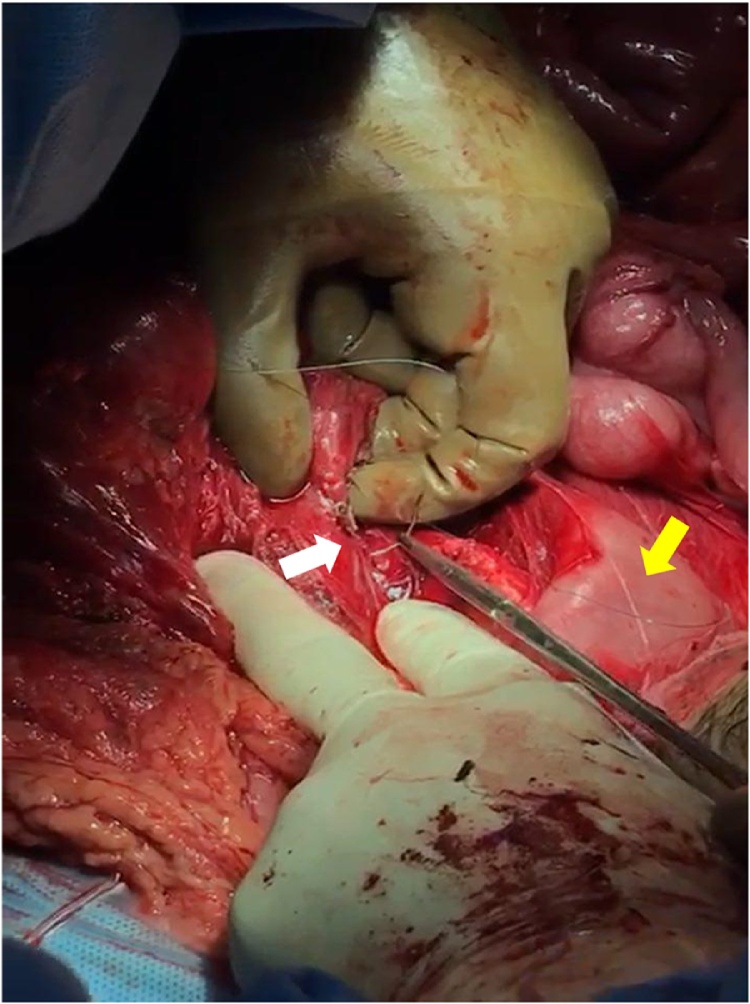
Repairing the SMV injury while applying proximal and distal pressure. White arrow points at the SMV injury. Yellow arrow points at the fourth part of the duodenum.

**Fig. 3:**
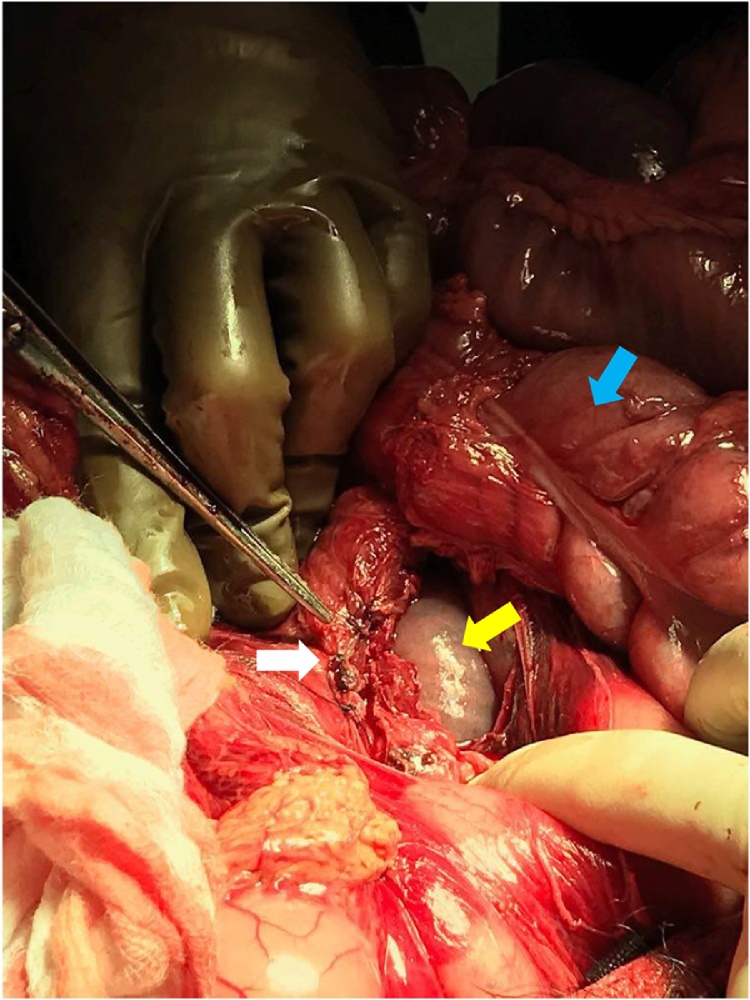
White arrow points at the SMV after repair. Yellow arrow points at the duodenum. Blue arrow points at the transverse colon.

**Fig. 4:**
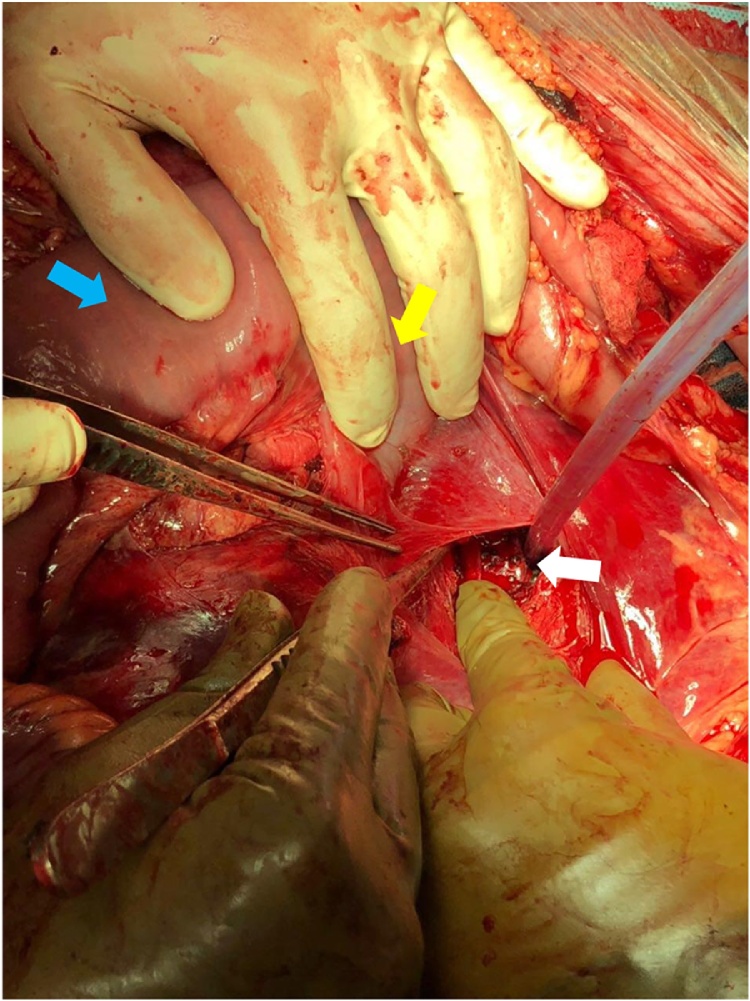
White arrow points at the pocket of pellets found in the retroperitoneum next to the aorta. Yellow arrow points at the duodenojejunal junction. Blue arrow points at the jejunum.

A twenty-six-year-old male presented to our emergency department after sustaining a self-inflicted gunshot wound to the abdomen thirty minutes prior to arrival. His past medical history was noted for bipolar disorder. Upon arrival by Emergency Medical Services (EMS), trauma code was activated. On primary survey, patient was hemodynamically unstable. His blood pressure was 90\65 mmHg, heart rate was 121 beats\minutes, and was maintaining his airway with a saturation of 98% on oxygen face mask with good bilateral air entry. He scored 14\15 in Glasgow Coma Scale (GCS), with bilaterally reactive three-millimeter pupils. Examination of the abdomen showed an entry wound midline, about ten centimeters above the umbilicus with no exit wound on logrolling. Focused Assessment with Sonography for Trauma (FAST) was positive for free fluid in Morrison’s pouch, spleno-renal and suprapubic regions. Patient received two liters of normal saline, two units of Packed Red Blood Cells (PRBC) class I, and one gram of Tranexamic acid. After that, the patient was intubated, with trauma line inserted, and immediately pushed to the Operating Room (OR) as first-level emergency case within fifteen minutes. A midline exploratory laparotomy revealed large amount of hemoperitoneum and active bleeding which was controlled by packing all four-quadrants of the abdomen. One bleeder was identified to be coming from the lesser curvature, most likely from the right gastric vein, which was controlled with a figure-of-eight PDS stich. After opening the lesser sac, there was another expanding hematoma posteriorly. Decision was made to kocherize the duodenum to reach the retroperitoneum, where another two bleeders were identified. One of which was from one of the supra-renal tributaries of the Inferior Vena Cava (IVC) at its lateral wall and the other coming from the SMV just inferior to the pancreas (Fig. 1). The site of SMV injury was in the mid-third point, around 3–5 cm from the IVC. The SMV injury was less than 1 cm in size, with a low-flow bleeding. Therefore, we cleared the bleeding vessel above and below the injury, identified the lesion, applied proximal and distal pressure, and a couple of stiches were taken in a figure-of-eight manner using PDS sutures (Fig. 2, Fig. 3). Then, we released proximal and distal pressure, and hemostasis was effectively achieved with our stitch. Multiple pellets were found, and we were able to remove around seventy-five of them. Exploration of major vessels using Mattox and Kocher maneuvers revealed a pocket of pellets next to the aorta, without other injuries (Fig. 4). Therefore, we proceeded with irrigation and partial closure of the abdomen with a Bagota’ bag. A total of eight units of PRBC and three units of Fresh Frozen Plasma (FFP) were given during the operation. The patient was shifted to the Surgical Intensive Care Unit (SICU), and post-operative CT scan and CTA run-off were done, showing patent vessels and no extravasation of contrast, with only minimal irregularity at the IVC injury site. The patient was taken back to the OR in less than forty-eight hours for a second-look laparotomy, which showed no active bleeding or other injuries. There was a concern for bowel viability, however, there was good supply as there was no ischemia or dusky bowel. Therefore, the packs were removed, irrigation and permanent closure of the abdomen were performed, and patient was sent back to the SICU. Two days later, the patient was extubated and started on oral feeding. Psychiatry consultation was made, and the patient was started on Olanzipine and Valproic acid. Close observation of the patient’s safety was ensured, with suicide precautions and one-to-one monitoring. The patient was then transferred to the general surgical ward the following day where he was observed daily by both the psychiatry and surgery teams. After two weeks, the patient was transferred to a local hospital specialized in mental health. Last follow up of the patient was in the surgery clinic, after one month from his first presentation, where he was in good health with no active issue.

Modified and reprinted with permission from [7].

## 3 Discussion

Abdominal vascular injuries are generally graded using the American Association for the Surgery of Trauma–Organ Injury Scale (AAST-OIS) for abdominal vascular injuries. SMV injuries are considered as grade III while both supra-renal and infra-renal IVC injuries are grade IV. Previous literature suggests a correlation between the grade of injury and mortality rates. According to Asensio et al., mortality rate for grade III injuries was reported to be 32%, and for grade IV mortality reaches 65% [4]. Specifically, penetrating abdominal trauma with injuries to the SMV, IVC and its tributaries are extremely lethal, and require a high index of suspicion for early recognition and management. Asensio et al. also reported that morality rates in traumatic IVC injuries were 75%, and SMV injuries carried a 57% mortality rates [8]. In our case, there are multiple aspects, which might have played a significant role in this patient’s favorable outcome. A major factor was owing to the anatomical accessibility of the injury and absence of associated injuries [3,8]. In addition to the patient’s baseline health status being young, fit, and not suffering from any medical co-morbidities which might have compromised his outcome otherwise. Most importantly, rapid response when faced with such injuries is crucial, by having a mature and developed hospital system that deals with trauma efficiently. It is also worth mentioning the importance of performing the surgery in stages with such injuries. The first time the patient was taken to the OR was mainly for stabilization and damage control surgery. Temporary closure using prosthetic abdominal wall closure allows for a second look to check for any missed injuries which might not have been discernible the first time to repair them, and then permanent closure of the abdominal wall. The choice of surgical treatment options between ligation and repair in SMV injuries remains a controversial topic in the literature. One study reports a 15% mortality rate after ligation compared with 36% mortality rate after primary repair [9]. IVC thrombosis is a rare but a well-recognized complication after surgical repair, which mandates imaging and close monitoring post-op, which was not there in our case.

## 4 Conclusion

SMV and IVC injuries are associated with high mortality rates and complications. Multiple factors have contributed to the patient’s favorable outcome. Having a mature system with a rapid trauma response allows for the best outcomes in such injuries. The principle of damage control surgery in trauma is an effective strategy to stabilize the patient and rule out other injuries which might not have been discernible initially.

## Conflict of interest

None.

## Sources of funding

None.

## Ethical approval

Our case report did not require an ethical approval.

## Consent

Written informed consent was obtained from the patient for publication of this case report and accompanying images. A copy of the written consent is available for review by the Editor-in-Chief of this journal on request.

## Author contribution

Nada Alhassan: literature review, acquisition of data, writing the manuscript.

Saad Alsaawy: editing of the manuscript, performing the surgery.

Ibrahim Albabtain: most responsible physician (MRP), performing the surgery, editing the manuscript.

All authors have reviewed and approved the final manuscript.

## Registration of research studies

Nothing to declare.

## Guarantor

Ibrahim Albabtain.

## Provenance and peer review

Not commissioned, externally peer-reviewed.
